# HLA, Immune Response, and Susceptibility to COVID-19

**DOI:** 10.3389/fimmu.2020.601886

**Published:** 2021-01-08

**Authors:** Fataneh Tavasolian, Mohsen Rashidi, Gholam Reza Hatam, Marjan Jeddi, Ahmad Zavaran Hosseini, Sayed Hussain Mosawi, Elham Abdollahi, Robert D. Inman

**Affiliations:** ^1^ Spondylitis Program, Division of Rheumatology, Schroeder Arthritis Institute, University Health Network, Toronto, ON, Canada; ^2^ Department of Pharmacology, Faculty of Medicine, Mazandaran University of Medical Sciences, Sari, Iran; ^3^ Basic Sciences in Infectious Diseases Research Center, School of Medicine, Shiraz University of Medical Sciences, Shiraz, Iran; ^4^ Endocrinology and Metabolism Research Center, Shiraz University of Medical Sciences, Shiraz, Iran; ^5^ Department of Immunology, Faculty of Medical Sciences, Tarbiat Modares University, Tehran, Iran; ^6^ Medical Sciences Research Center, Ghalib University, Kabul, Afghanistan; ^7^ COVID-19 Directorate, Ministry of Public Health, Kabul, Afghanistan; ^8^ Department of Medical Immunology and Allergy, School of Medicine, Mashhad University of Medical Sciences, Mashhad, Iran; ^9^ Department of Medicine and Immunology, University of Toronto, Toronto, ON, Canada

**Keywords:** Coronavirus Disease 2019, human leukocyte antigens, original antigenic sin, immune response, vaccine design

## Abstract

The severe acute respiratory syndrome caused by Coronavirus 2 (SARS-CoV-2) that appeared in December 2019 has precipitated the global pandemic Coronavirus Disease 2019 (COVID-19). However, in many parts of Africa fewer than expected cases of COVID-19, with lower rates of mortality, have been reported. Individual human leukocyte antigen (HLA) alleles can affect both the susceptibility and the severity of viral infections. In the case of COVID-19 such an analysis may contribute to identifying individuals at higher risk of the disease and the epidemiological level to understanding the differences between countries in the epidemic patterns. It is also recognized that first antigen exposure influences the consequence of subsequent exposure. We thus propose a theory incorporating HLA antigens, the “original antigenic sin (OAS)” effect, and presentation of viral peptides which could explain with differential susceptibility or resistance to SARS-CoV-2 infections.

## Introduction

More than 190 countries have experienced a recent COVID-19 pandemic, especially China, South Korea, Italy, Iran, Spain, France, the United Kingdom, and growing numbers from the United States. However, Africa, with a population of >1.2 billion people, has had a comparatively low percentage of COVID-19 deaths, especially in the malaria-endemic region (1–6). There have been several hypotheses about such unexpected findings, including a relative lack of testing and documentation. The confirmation studies have suggested that most of these countries have close ties with China in terms of trade, migration, or commerce. All of these may play a role, yet it has also been proposed that several African nations more vigorously implemented public health policies than many other countries. Another consideration is that epidemic preparedness may be much higher, with prior experience with Ebola, human immunodeficiency virus (HIV), tuberculosis (TB), etc. The strong burden of endemic infectious diseases in sub-Saharan Africa, and ongoing outbreaks of Lassa fever and Ebola in Nigeria and Congo, suggest an unpredictable and unusual response to COVID-19 (5, 7). This background illustrates the need to utilize a range of mitigation approaches even during the pandemic to establish a broad-based response. Our hypothesis emphasizes that HLA antigens and the OAS phenomenon could be important determinants for outcomes following SARS-CoV-2 infection or vaccination (8, 9).

Coronaviruses are a category of respiratory viruses that can cause infections ranging from the common cold to Middle-East respiratory syndrome (MERS) and severe acute respiratory syndrome (SARS). An increasing body of literature reveals that these coronaviruses are originally zoonotic, targeting the lower respiratory tract, and causing potentially lethal inflammation in extra-pulmonary organs (10). Within two decades, there have emerged three highly pathogenic and deadly human coronaviruses: SARS-CoV, MERS-CoV, and SARS-CoV-2 (11). What started as a novel outbreak of atypical viral pneumonia in December 2019 in Wuhan, China is now officially recognized as COVID-19, with the causative virus classified as SARS-CoV-2, which expresses a genomic homology of about 80% to SARS-CoV, and lesser homology (50%) with MERS-CoV. Researchers have discovered that cross-reactive antibodies react with, but do not confer cross-protection against, the SARS-CoV-2 receptor-binding domain (RBD) and non-RBD domains, nor is there cross-neutralization between SARS-CoV and SARS-CoV-2 are uncommon (12–15).

SARS-CoV-2 is a single-strand RNA coronavirus composed of four main structural proteins: spike (S), envelope (E), nucleocapsid (N), and membrane (M) proteins that induce extreme respiratory disease and an aggressive pneumonia-like infection (16). Clinical studies have demonstrated that fever, exhaustion, dry cough, shortness of breath, and acute respiratory distress syndrome (ARDS) are predominant clinical manifestations. Several research studies have confirmed that the severity of the infection has been correlated with lymphopenia and the development of a cytokine storm (17).

## Association Between HLA and COVID-19

In humans, the HLA system orchestrates immune regulation. The research effort, therefore, aims to identify the mechanisms that are potentially responsible for activating an immune response to SARS‐CoV‐2, including the role of HLA alleles in affected individuals (18). It is recognized that T-cell receptors recognize the conformational structure of the antigen binding-grove in the HLA molecule along with the accompanying antigen peptides. Thus, particular HLA haplotypes are associated with distinct genetic predispositions to disease (9, 19, 20). The repertoire of the HLA molecules composing a haplotype is thought to contribute to survival during evolution. As a result, it is advantageous to have enhanced binding capabilities of HLA molecules for viral peptides on the surface from novel viral infections, such as SARS-CoV-2, on the cell surface of antigen-presenting cells (9, 20–22).

We speculate that population HLA variability in a population could be correlated with COVID-19 incidence since HLA plays such a crucial role in the immune response to pathogens and the development of infectious diseases. The HLA system affects clinical outcomes in multiple infectious diseases, including HIV and SARS (23, 24). For the latter, population studies observed correlations between certain HLA alleles and the incidence and severity of SARS (24–26). HLA-B*07:03, B*46:01, DRB1*03:01, DRB1 *12:02 alleles were correlated with SARS susceptibility (27, 28). The SARS-CoV-2 sequence displays considerable homology with SARS, but the two viruses do have distinct variations (29). Therefore, it will require further investigation to incorporate HLA alleles when analyzing COVID-19 outcomes (27, 30, 31). The SARS-related susceptibility alleles were not shown to occur in COVID-19 patients at a significantly different level after p-value correction in the analysis performed by Wei Wang et al. in May 2020 (32).

Host genetic variability may help explain the multiplicity of immune responses to a virus within a community. Knowing how variability in HLA can impact the progression of COVID-19, in particular, may help distinguish individuals at higher risk for the disease.

In a study conducted by Benlyamani et al. in critically ill patients, results indicate downregulation of HLA-DR molecules in circulating monocytes, which, based on profound lymphopenia and other functional differences, create immunosuppressed conditions for host response (33).

An *in silico* analysis of viral peptide-major histocompatibility complex (MHC) class I binding affinity was conducted by Nguyen et al, which revealed that HLA-A*02:02, HLA-B*15:03, and HLA-C*12:03 effectively presented a larger amount of peptides whereas A*25:01, B*46:01, C*01:02 were the least efficient for of SARS-CoV-2 peptide presentation (30). Iturrieta-Zuazo et al. indicate that Class I HLA molecules with a better theoretical capacity to bind SARS-CoV-2 peptides were found in patients with mild disease and showed higher heterozygosity as compared with moderate and severe disease (34).

Thus the genetic variability of the MHC molecules can affect the susceptibility and severity of SARS-CoV-2 (35). One potential genetic contributor to the lower incidence of SARS-CoV-2 in Africa may be the occurrence of different HLA alleles in Africa compared to other regions. HLA alleles, particularly MHCI, are major elements of the presentation system for viral antigens and have been shown to impart differential viral resistance and disease intensity. Specific HLA genotypes can stimulate the T cell-mediated anti-viral response differently and could possibly alter the symptoms and transmission of the disease (36). HLA-B*46:01 is expected to have the fewest possible binding peptides for SARS-CoV-2, indicating that individuals with this allele could be especially vulnerable to COVID-19, as had previously been seen for SARS. HLA-B*15:03, on the other hand, demonstrated the greatest ability to present highly conserved SARS-CoV-2 peptides shared among common human coronaviruses, indicating that this allele may allow cross-protective T-cell dependent immunity (30). This is a more intriguing mechanism, as HLA-B*1503 appears to be prevalent in West Africa and most countries with high endemic malaria in the World Health Organization(WHO) African Region (37).

The HLA-viral interaction is complex. While HLA-B27 seems to confer relative resistance to Hepatitis C virus(HCV) and HIV (38, 39), it may confer susceptibility to malaria (40), since in Africa HLA-B27 prevalence seems to decrease in malaria-endemic populations. This could be related to the hypothesis of HLA-mediated host peptide presentation since serious COVID-19 disease is particularly unusual in malaria-endemic populations. In particular, a substantial correlation for HLA‐DRB1*15:01, ‐DQB1*06:02, and ‐B*27:07 was observed in research conducted Novelli et al. in a group of 99 Italian patients affected by a severe or extremely severe course of COVID‐19, although considering the limited sample size, there is a chance of false-positive detection (41). In the case of influenza, A (flu), HLA-B27-related immunodominance is endoplasmic reticulum aminopeptidase (ERAP-1)-dependent. This is not the case for HLA-B7 immunodominance the epitope of the HLA-B27 immunodominant influenza nucleoprotein (NP) 383–391 is formed as a 14-mer N-terminally stretched until ERAP-1 trims it. In flu-infected B27/ERAP-/- mice, the CD8+ T cell reaction to the B27/NP383–391 epitope is significantly reduced in the absence of ERAP1 As the correlation between ERAP-1 and ankylosing spondylitis (AS) is seen exclusively in HLA-B27+ patients, it appears that creating the B27-related immunodominant peptide of the flu virus depends on ERAP-1, but this is not the case for HLA B-7 (42). Whether this ERAP-HLA interaction is applied to coronaviruses as well is unknown. Currently, we have limited data on whether B27+ AS patients are more, or less, susceptible to COVID-19 (43).

## Original Antigenic Sin and Immune Response in COVID-19

Coronaviruses belong to the family Coronaviradae, order Nidovirales, that can be further categorized into four major lines (α-, β-, π-, and δ-coronaviruses). Several α- and β-coronaviruses induce mild respiratory infections and common cold symptoms in humans, while some are zoonotic and infect birds, pigs, bats, and other animals. Similar to SARS-CoV-2, two other coronaviruses, SARS-CoV, and MERS-CoV, also caused large disease outbreaks with high mortality levels (10%–30%) and substantial social effects. Comparison of the SARS-CoV-2 protein sequence with the sequences SARS-CoV, MERS-CoV, and bat-SL-CoVZXC21 revealed a high degree of homology between SARS-CoV-2, bat-SL-CoVZXC21, and SARS-CoV, but with a more restricted similarity to MERS-CoV. Grifoni et al. proposed that SARS-CoV-2 may be potentially interpreted as the inevitable outcome of an antigenic shift from SARS-CoV since these viruses share around 80% of their genome and almost all the encoded proteins (14).

Identifying how pre-existing immunity may dictate the development of antibodies to conserved SARS virus epitopes is important for the creation of new SARS-CoV-2 virus vaccines. Two features that have been found during the SARS-CoV-2 pandemic demand attention. First, the lack of clinical symptoms of infection in children (16, 44), Secondly, the early onset of IgG in serum (45, 46). From the viewpoint of the host immune response, such an early increase of specific IgG is thought to represent a secondary immune response when the memory of a cross-reactive antigen is present, as may occur from an earlier coronaviral infection.

The key question then emerges whether or not such cross-reactive antibodies defend against the new virus. The worst-case scenario would be for such cross-reactive memory antibodies against similar coronaviruses not only to be non-protective but also to intensify infection (47). A significant discovery, however, is that cross-reactivity in antibody binding to spike proteins in SARS-CoV-2 and SARS-CoV infections is widely observed, suggesting that antibodies to conserved spike antigens are common. Cross-neutralization of the virus species is however an uncommon phenomenon (12). In a study conducted by Ju et al. the anti-SARS-CoV-2 antibodies and the convalescent plasma did not cross-react with the RBD of the viral spike protein of SARS-CoV or MERS-CoV, although there was substantial cross-reactivity to their trimeric spike proteins (48). But the plasma antibodies did cross-reaction with antigens in the SARS-CoV and MERS-CoV spikes, which did not result in virus neutralization (47, 48).

**Figure 1:**
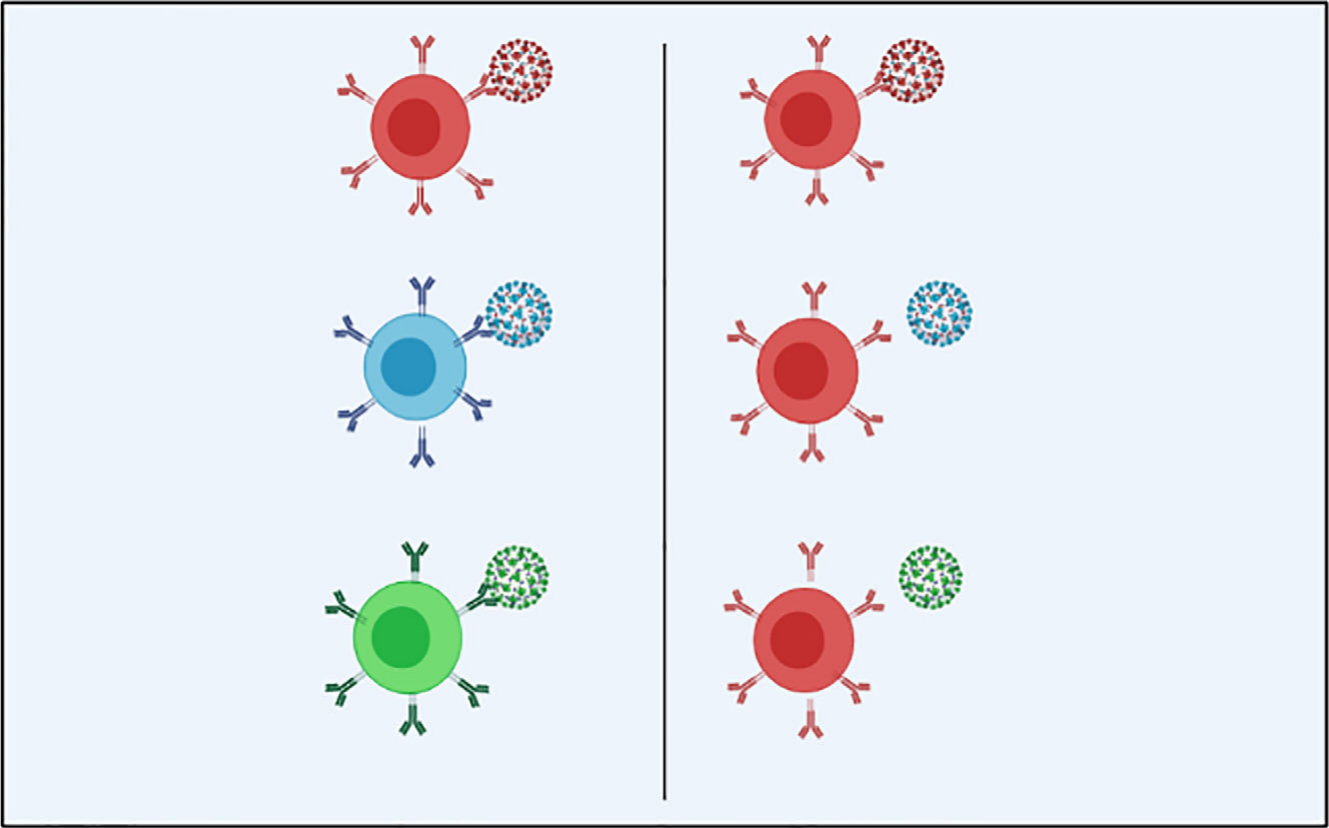
In the optimal immune response (on the left) against SARS-CoV-2 and its antigenic variants, the particular adaptive immunity is often associated with (color matching) the symbolic antibody and the spike proteins that cover the outer surface of the virion; Original antigenic sin explains the propensity of the immune response to use immunological memory that relies on the previous infection when a new slightly altered strain of the foreign pathogen is identified. As we see in the OAS model (right), the particular adaptive immune response is only installed against the original virus and is not used to combat the mutated forms of the virus, leading to a less specific and less efficient maladaptive response (Created in BioRender.com) (8).

Original antigenic sin (OAS) refers to the activation of a more vigorous immune reaction to the priming vs an immunogenic boost that itself attaches poorly, if at all, to antibodies s caused by the priming immunogen (**Figure 1**) (8, 49, 50). MHC diversity could be a contributor in humans for such an event (30).

### OAS and B Cells

Long-lived B memory cells, which persist in the body, develop during primary infection, and protect against subsequent infection. To produce antigen-specific antibodies, these memory B cells respond to particular epitopes on the surface of viral proteins and are capable of responding to infection faster than B cells react to novel antigens. This effect reduces the time it takes for subsequent infections to be resolved (51, 52).

A virus may demonstrate antigenic drift following primary and secondary infections, in which the viral epitopes are reshaped by natural mutation, enabling the virus to evade the immune system. When this occurs, the modified virus preferentially reactivates previously activated B memory cells of high affinity and stimulates the production of antibodies. Such antibodies may prevent the recruitment of higher affinity naive B cells that could generate more effective antibodies against the second viral challenge. This contributes to a less efficient immune response since it can take longer to clear recurrent infections. For the development and implementation of vaccines, OAS is of particular interest. The actual impact of OAS in dengue fever has important consequences for vaccine research. Whenever a response has developed to a dengue virus serotype, vaccination to a second serotype is unlikely to be successful, meaning that balanced reactions to all four virus serotypes should be developed in the initial formulation of the vaccine (53). However, in 2015 a new category of highly effective neutralizing antibodies was isolated, which were efficient against those four virus serotypes, boosting hope for the development of a universal dengue vaccine. In people that are regularly immunized, either by vaccinations or chronic infections, the specificity and efficiency of the immune response to new influenza strains are sometimes reduced (54). The effect of antigenic sin on protection, however, has not been well characterized and seems to vary for each vaccine with the particular pathogen, geographical region, and age.

## OAS and Cytotoxic T Cells

A similar pattern in CTLs is implicated in host immunity. With a second infection by another type of dengue virus, it has been seen that the CTLs tend to produce cytokines rather than inducing target cell lysis. As a consequence, the production of these cytokines is suggested to enhance vascular permeability and the destruction of endothelial cells is intensified (55).

Many researchers have attempted to formulate HIV and HCV vaccines based on the induction of host CTL response. The observation that original antigenic sin can distort the CTL response can shed light on understanding the restricted efficacy of such vaccinations. Viruses such as HIV are extremely variable and regularly undergo mutation. In such circumstances, a vaccine may fail to control HIV infection if the virus expresses slightly different epitopes compared to those in the viral vaccine due to original antigenic sin. In theory, the vaccine could exacerbate the infection by “trapping” the immune response into the initial, inefficient response to the virus (56).

Thus, an inadequate immune response to the mutated virus due to the OAS may generate a significant number of sub-neutralizing cross-reactive antibodies that enhance inflammation and may paradoxically promote virus entry into host cells (8). The intracellular presence of the pathogen activates a pyroptosis mechanism with the subsequent release of danger-associated molecular patterns (DAMPs) to trigger additional inflammatory cells, which in response release a great number of cytokines; which may be the basis of the “cytokine storm” identified in severe cases of COVID-19 (17, 57).

## Is OAS Harmful?

Consequentially, the OAS phenomenon can be both advantageous and detrimental to host defense. Perhaps the biggest influence of both the risks and benefits of OAS is an ever-present threat from strongly **pathogenic strains of zoonotic viruses** with pandemic potential. These are new pathogens for humans and it can be dangerous for any individual who does not have an element of pre-existing cross-protective immunity once a strain of this type appears. However, imprinting with a strain from the same group of phylogenies may protect against serious infection. Both the H1N1 pandemics of 1918 and 2009 have been accelerated by the reduced vulnerability of aging populations to cross-protection from antibodies generated to strains throughout childhood (58, 59). Also, heterosubtypic defense against highly pathogenic avian strains may rely on the year of birth, and hence the dominant strain during early life (60). This raises the possibility of a degree of protective immunity conferred by SARS-CoV and MERS-CoV-specific antibodies to decrease the susceptibility to SARS-CoV-2 in Africa by the higher prevalence of HLA-B*15:03, which has the highest strength to present strongly conserved SARS-CoV-2 peptides shared by common human coronaviruses. Resolving this interaction may allow cross-protective T cell immunity to be derived (8, 61).

These correlations were also found between immunodominant sequences [N protein from SARS-CoV-2/SARS-CoV and thrombospondin-anonymous-related protein (TRAP) from P. falciparum] and [S protein-SARS-CoV-2/SARS-CoV and predicted epitope in sporozoite surface protein -2 (SSP-2) from P. falciparum]. Individually, both epitopes could induce the response of CD8+ CTL by HLA-A*02:01 presentation (1, 2). Lesa et al. presumed that the memory of adaptive immunity elevated against the described TRAP immunodominant epitope could recognize peptide-HLA-A*02:01 complexes originating from SARS-CoV-2 infection in malaria-endemic regions, particularly in Africa, and modify the host immune system response. Of course, such an assumption needs further empirical testing to prove its validity and ascertain the strength of the primed response (1).

## Vaccine Design

The creation of an efficient subunit vaccine seems to be rather challenging in the presence of OAS and the potential adaptive mutation of SARS-CoV-2. Thus, an attractive option is to concentrate on an alternative method of vaccination that is capable of stimulating innate immunity instead of adaptive immunity. In children, where the immune system is immature and susceptible to challenge with new antigenic stimuli, innate immunity may be more effective, while adaptive immunity may play are larger role in the mature immune response in adults. This may relate to the observation that children rarely suffer fatal complications during the current COVID-19 pandemic (8, 62).

One of the representative processes of immune evasion of pathogens is to mask or alter pathogen molecules which are usually recognized by innate pattern recognition receptors. When developing vaccines against infectious agents, there is also a need to focus an immune response towards a particular conformational epitope (63). The approach is to develop vaccines that can provide epitope-specific immunofocusing and induce antibodies specifically targeting epitope (64). A specific monoclonal antibody could be used to shield the target epitope on the protein. The remaining uncovered surface proteins would then be altered to make them non-immunogenic. The epitope is eventually unprotected by eliminating the monoclonal antibody (64). The conserved immunodominant regions from Coronaviruses have implications for vaccine design against SARS-CoV-2 because of the OAS phenomenon. Research strategies must seek to maximize antibodies to conserved epitopes and induce broadly protective immunity against multiple strains. By utilizing an adjuvant vaccine, an increased cellular reaction will yield enhanced host protection benefits (65).

## Conclusion

The clinical course of infection with SARS-CoV-2 is strongly dependent on the relationship between the virus and the host immune system, in which the host HLA plays a central role in the activation and regulation of the immune response. There is scope for further study into the role of HLA in COVID-19, and epidemiological studies need to focus on HLA profiles as host immune determinants. Such studies should include HLA typing of COVID-19 patients, both to unravel the complexity of the disease response and also to inform customized therapies. In addition, a prior history of coronavirus infection in the patient can be relevant to the magnitude of the immune response to the current SARS-CoV-2 infection, a phenomenon referred to as “original antigenic sin”. This concept refers to cross-reacting immunity due to past infections of similar coronavirus strains, which must be considered in interpreting immune responses to infections and vaccinations.

## Author Contributions

All authors listed have made a substantial, direct, and intellectual contribution to the work and approved it for publication.

## Conflict of Interest

The authors declare that the research was conducted in the absence of any commercial or financial relationships that could be construed as a potential conflict of interest.

## Abbreviations

SARS-CoV-2, Severe Acute Respiratory Syndrome Coronavirus 2; COVID-19, Coronavirus Disease 2019; HLA, Human Leukocyte Antigen; OAS, Original Antigenic Sin; HIV, Human immunodeficiency virus; TB, Tuberculosis; MERS, Middle-East respiratory syndrome; SARS, Severe Acute Respiratory Syndrome; S, spike; E, envelope; N, nucleocapsid; M, membrane; ARDS, Acute Respiratory Distress Syndrome; WHO, World Health Organization; HCV, Hepatitis C virus; Flu, influenza A; ERAP, Endoplasmic Reticulum Aminopeptidase; NP, nucleoprotein; AS, Ankylosing Spondylitis; RBD, Receptor-Binding Domain; MHC, Major Histocompatibility Complex; CTL, cytotoxic T cells; DAMPs, Danger-Associated Molecular Patterns; TRAP, Thrombospondin-Anonymous-Related Protein; SSP-2, Sporozoite Surface Protein -2.
